# Supplementary material for: Chromothripsis during telomere crisis is independent of NHEJ, and consistent with a replicative origin
Source: Genome Res. 2019 May;29(5):737–49. doi: 10.1101/gr.240705.118 (PMC6499312; doi:10.1101/gr.240705.118)
Supplement: Supplemental Material [file supp_gr.240705.118_Supplemental_file_1.zip › contigs/annotated_contigs/DB50/contig.2.DB50_length_301_mean_cov_1.90365448505.docx]

**DB50_length_301_mean_cov_1.90365448505**

```
CCCTGAAATGATATTTCTCTTATCAGTGGATCTTCCCCCTGGCTGCACTTCAGAATCCCGGGGGAGCTTCAGCAAATACCACGGCCGGG
 >chr10:126552335-126552538 + E=5e-105 p=1e-02
GCCCAGCCCTGGGGCTTCTGGAGTAGGGCTGGGGCCCATCCCCGGGGCTTCTGGAGCAGGCCTGGGGCCACTGGGAGGGTTCAAGAGCT

CAGCAGGTAATTTTATTTATTTTTT|TATTTTTAGTTTTTTTTTTACTCTGGCTTTAGTAT|AGTGGCACGATCTCAGCTCACTACAGC
                                                             >chr10:126552573-126552636
CTCCACCTTCTGGGTTTAGGCGAGTCTTCTGCCTTA + E=2e-14
```
